# The Dorsal Rather than Ventral Pathway Better Reflects Individual Syntactic Abilities in Second Language

**DOI:** 10.3389/fnhum.2016.00295

**Published:** 2016-06-14

**Authors:** Kayako Yamamoto, Kuniyoshi L. Sakai

**Affiliations:** ^1^Department of Basic Science, Graduate School of Arts and Sciences, The University of TokyoTokyo, Japan; ^2^Japan Society for the Promotion of ScienceTokyo, Japan; ^3^Core Research for Evolutional Science and Technology (CREST), Japan Science and Technology AgencyTokyo, Japan

**Keywords:** diffusion MRI, white matter, dorsal and ventral pathways, language acquisition, syntax, monozygotic twins

## Abstract

The left inferior frontal gyrus (IFG) has been reported to be critically involved in syntactic processing, not only in first language (L1), but in second language (L2). Indeed, the leftward lateralization of the IFG has been shown to be correlated with the performance of a syntactic task in L2. Given that posterior language-related regions are systematically connected with the left IFG, the next question is which of the dorsal and ventral pathways is more critical to the individual syntactic abilities in L2. Here we used diffusion magnetic resonance imaging (MRI) and tractography with newly developed semi-automatic methods of defining seeds and selecting regions of interest (ROIs). We calculated mean thickness and fractional anisotropy (FA) in each ROI for the arcuate fasciculus (Arcuate) of the dorsal pathway, as well as for the inferior fronto-occipital fasciculus (IFOF) of the ventral pathway. In Experiment I, we performed partial correlation analyses between FA and the accuracy of the syntactic task, removing the effects of the accuracy of a spelling task, gender, and handedness. Among the two pathways in each hemisphere, only FA of the left Arcuate was significantly correlated with individual accuracy of the syntactic task. In Experiment II, we recruited monozygotic twins and examined to what extent their L2 abilities and their structural properties were similar. Within twin pairs, the highest significant correlation was observed for reaction times of the spelling task, while the correlation for the accuracy of the syntactic task was marginal; these two correlation coefficients were significantly different. Moreover, the thickness of the left Arcuate was highly correlated within pairs, while its FA, as well as the thickness/FA in the ventral pathways, was not significantly correlated. The correlation coefficient for the thickness of the left Arcuate was significantly larger than that of the left IFOF. These results suggest that the thickness of the left Arcuate is more associated with the shared genetic/environmental factors, whereas both of mutually correlated FA in the left Arcuate and individual syntactic abilities in L2 may be less prone to these shared factors.

## Introduction

It has been widely accepted that the cortical regions supporting language functions are mainly connected by dorsal and ventral pathways (Hickok and Poeppel, 2007; Friederici, 2011). The recent development of diffusion magnetic resonance imaging (MRI) techniques has opened an avenue for the structural studies of fiber bundles in a non-invasive manner, by enabling, for example, the assessment of each bundle’s fractional anisotropy (FA)—the degree of fiber organization and/or myelination. The bundle’s volume or thickness is another structural property independent of FA, since it is possible that the tract becomes thicker even when the tract has lower FA. FA has been reported to increase in white matter underlying the intraparietal sulcus after a 6-week training of a visuo-motor skill (Scholz et al., 2009), as well as in the genu of the corpus callosum during a 9-month training of a second language (L2; Schlegel et al., 2012). These studies have induced researchers to examine a correlation between FA and performance metrics. In order to reveal the functional roles of the language-related pathways, it would be important to separate syntactic or semantic abilities from other general skills, and to further identify which structural properties of dorsal and/or ventral pathways actually reflect individual differences in these abilities.

When tracking pathways in an individual brain with diffusion MRI, we should assess the structural properties of a certain tract with caution, because in some studies not all of the *right* arcuate fasciculus (Arcuate), which overlaps with the superior longitudinal fasciculus (SLF), were reconstructed (Catani et al., 2007; Lebel and Beaulieu, 2009; Thiebaut de Schotten et al., 2011; López-Barroso et al., 2013). Possible causes of such uncertainty may be the limitations of deterministic tracking algorithms (Yeatman et al., 2011), and/or other methods for tracking bundles—for example, starting points (i.e., seeds) were drawn manually in many studies, making the strict inclusion/exclusion of certain fibers difficult. The use of probabilistic tracking algorithms, as well as a reliable and objective method for seed definition, is thus necessary. Another difficulty in examining structural properties of individual brains is that macroscopic differences may depend on a number of structural factors, such as the degrees of arching and branching, as well as individual variations in the shape and size of tracts, especially near the gray matter (Catani et al., 2005; Anwander et al., 2007). One approach for focusing on areas of small inter-subject variability is to select regions of interest (ROIs) that exclude potentially variable branching regions (Tsang et al., 2009; Yeatman et al., 2011). Although there is another strategy of Tract-Based Spatial Statistics (TBSS), in which voxels in the center of fiber bundles with the highest FA (i.e., the “mean FA skeleton”) are examined (Smith et al., 2006), it has been argued that the mean FA skeleton is a very limited portion at the center (Van Hecke et al., 2010; Bach et al., 2014). Selecting ROIs has an advantage over TBSS, such that the thickness (or volume), i.e., another structural property, can be obtained at a full-width cross-section of a fiber bundle. Here we employed this ROI approach, and tried to improve the method of determining the size and position of ROIs objectively.

In the present diffusion MRI study with probabilistic tracking, we developed the following semi-automatic methods, and minimized the manual procedures. Seeds were defined in a semi-automatic and consistent manner, and the tracts were successfully reconstructed in both hemispheres for every participant. We then selected a ROI in each tract at the portion with the most uniform *thickness*, thereby excluding the branching or curved portions. The ROI size for each pathway was also optimized to minimize the individual variances of thickness. By focusing on these ROIs, we were able to reliably examine whether individual variances of FA, independent of thickness, in the language-related pathways reflect the individual linguistic abilities. In the present study, we focused on individual differences in L2 acquisition, because varieties in L2 abilities are usually larger than those in first language (L1).

Here we recruited Japanese students who had learned English as L2, and assessed their individual syntactic abilities using a syntactic error-detection (Syn) task in English. The syntactic errors were basically related to the argument structures of English verbs. In some linguistic theories, it is assumed that “lexical entries contain at least some syntactic information, in addition to the phonological and semantic information that surely must be present” (Chomsky, 1995). Previous functional MRI studies have employed tasks for argument structures, revealing the selective activations for syntactic decisions in the left inferior frontal gyrus (IFG; Suzuki and Sakai, 2003; Raettig et al., 2010). Magnetoencephalography (MEG) studies have also shown selective responses in the left IFG to verbs depending on argument structures, thereby controlling the effects of lexico-semantic factors (Iijima et al., 2009; Inubushi et al., 2012; Iijima and Sakai, 2014). Cortical regions other than the left IFG may also contribute to syntactic processing. It has been reported that damage to the left temporo-parietal cortex including the middle/superior temporal gyri and the angular gyrus is related to the decreased performances of sentence-picture matching tasks with several sentence types (e.g., active, passive, and cleft sentences; Dronkers et al., 2004; Thothathiri et al., 2012; Magnusdottir et al., 2013). However, words used in these tasks may also have pragmatic or semantic effects, and the functions of these regions may be explained by these additional effects. Returning to the functions of the left IFG in syntactic processing, we have clarified its critical involvement not only for L1, but for L2. In our previous functional MRI study (Sakai et al., 2009), we used the same tasks as in the present study, including a spelling error-detection (Spe) task to control semantic and related cognitive factors. We have demonstrated that the individual accuracy of the Syn task was positively correlated with activations of the dorsal triangular part (F3t) of the left IFG. Moreover, our voxel-based morphometry (VBM) study with the same tasks showed that the accuracy of the Syn task, but not of the Spe task, was correlated with the leftward lateralization of a single region’s volume in the F3t (Nauchi and Sakai, 2009). These functional MRI and VBM studies have provided a consistent view that the individual differences both in the function and anatomy of the left IFG were related to the individual syntactic abilities. Regarding connectivity including regions adjacent to the left IFG, resting-state connectivity between the left anterior insula/frontal operculum and the left superior temporal region has been reported to show a positive correlation with other L2 abilities including lexical retrieval (Chai et al., 2016). In such resting-state connectivity, however, the *functional* roles of those pathways remain unknown. It would be important to examine the correlation between behavioral metrics and any properties of anatomically or functionally identified pathways. Given our previous studies suggesting the critical involvement of the left IFG to syntactic processing in L2, a next question is to clarify which of structural properties of white matter fibers connecting the left IFG reflect individual syntactic abilities.

Recent diffusion MRI studies have anatomically identified several dorsal and ventral pathways connecting inferior frontal regions with other cortical regions supporting language functions (Catani et al., 2012). Our recent study, using functional/diffusion MRI as well as functional connectivity analyses, showed that the left dorsal pathway including the Arcuate/SLF transmitted information from the left IFG to the supramarginal gyrus, where syntax-selective activations were observed (Ohta et al., 2013). On the other hand, a lesion study reported that disconnection in either the dorsal or ventral pathway was associated with increased errors in a sentence-picture matching task, suggesting that both the dorsal and ventral pathways are critical for syntax (Griffiths et al., 2013). In order to reveal the roles of connectivity including the left IFG in L2 acquisition, it is thus important to separate syntactic abilities from other linguistic or general ones, and to further clarify whether the structural properties of the dorsal and/or the ventral pathways actually reflect individual syntactic abilities. In the present study, we focus on the Arcuate of the dorsal pathway, as well as the inferior fronto-occipital fasciculus (IFOF) of the ventral pathway, and hypothesize that structural properties of the *left* Arcuate reflect the individual syntactic abilities in L2. We recruited native Japanese speakers, who had studied English as L2 for about 5 years, and assessed their syntactic abilities in L2 based on the accuracy of the Syn task (Experiment I).

We further examined to what extent L2 abilities and the brain structures are associated with factors that are shared among individuals (e.g., a particular educational environment), and with those that are not shared among individuals (e.g., individual aptitudes for multiple languages). Because monozygotic twins share genetic and some environmental factors, twin studies can be used to explore the extent to which brain structure or function is influenced by shared genetic and environmental determinants. A monozygotic twin study has reported that the volume of each of four lobes was strongly affected by shared genetic/environmental factors, while the sulcal/gyral curvature and the surface-to-volume ratio were more prone to be affected by non-shared factors (White et al., 2002). Monozygotic twins have also been studied to reveal the influence of shared factors on sulcal patterning using a graph matching method (Im et al., 2011). A possible direction of research includes the following two steps: (i) identifying structural properties and behavioral performances that reflect specified functions (as in our Experiment I); and (ii) examining the correlations within monozygotic twin pairs for each of the structural and behavioral parameters to explore the extent of their dependence on shared factors. We assessed the behavioral correlations within monozygotic twin pairs (Experiment II), thereby examining the extent to which the accuracy and reaction times (RTs) in the Syn/Spe tasks in L2, as well as a verbal fluency in L1, are associated with shared factors; we do not intend to dissociate genetic and environmental factors here. We also examined whether the white matter properties, i.e., the thickness and FA, were associated with shared factors. Our present study with monozygotic twins, together with appropriate methods to examine the structural basis of syntactic abilities, should shed new light on the neural framework underlying individual differences in L2 acquisition.

Here we conducted diffusion MRI acquisition and fiber tracking by using *q*-ball imaging. The *q*-ball imaging technique, a *q*-space analysis of diffusion MRI with a relatively high *b*-value (e.g., 4000 s/mm^2^), was developed for improved reconstruction of the fiber tracts (Tuch, 2004). On the other hand, the *b*-value was often lowered to 1000 s/mm^2^ for a better estimation of FA (Jones and Basser, 2004). For high *b*-values, the assumption that the diffusion signal decay is monoexponential may not be suitable (Clark and Le Bihan, 2000), but such a difference in FA is smaller with a 3 T scanner than with a 1.5 T scanner (Chung et al., 2013). In the present study we used a 3 T scanner and conducted a pilot experiment to examine the effects of imaging parameters.

## Materials and Methods

### Experiment I

#### Participants

We recruited senior high-school students in their fifth academic year at the Secondary Education School attached to the Faculty of Education of the University of Tokyo, to which twins are preferentially admitted for educational research. We set the following basic inclusion criteria: (i) right-handedness as assessed by the Edinburgh inventory (Oldfield, 1971); (ii) no history of neurological or psychiatric diseases; and (iii) native Japanese speakers whose age of first exposure (AOE) to English in formal education was 12 or 13 years old (a condition met by the majority of students in this school). As regards the first criterion, participants with a negative laterality quotient (LQ) or those with a potential history of change in handedness were dropped, resulting in a relatively strong right-handed population (LQ > 50 in most cases). In Experiment I, for each twin pair who met these criteria, the one exhibiting the higher score for the Spe task was entered into the analysis to avoid a double count of twins with potentially similar characteristics. There were 47 participants who met these conditions.

We set two additional criteria in Experiment I: (iv) longer RTs for Syn than for Spe; and (v) performance accuracy in the upper half of participants for Spe (80% or higher). The fourth criterion was necessary because shorter RTs for Syn indicated the possible abandonment of the more demanding task; two participants were dropped for this reason. Given that the participants in the present study did not receive any training sessions, it was necessary to set the fifth criterion in order to match the task accuracy of Syn [both mean and standard deviation (SD)] with that *after the training* reported in our previous studies (Nauchi and Sakai, 2009; Sakai et al., 2009); 19 participants were dropped for this reason. When the fifth criterion was not applied, the accuracy of Syn for 20 out of the 47 participants was lower than 55% (i.e., near the level of chance); such a population was not appropriate for examining individual syntactic abilities. Twenty-six participants met the five criteria, and demographic details of these participants are shown in Table 1: age, AOE to English, duration of exposure (DOE) to English, and LQ. Written informed consent was obtained from all participants as well as from their guardians. The study was approved by the Secondary Education School and by the Institutional review board of the University of Tokyo, Komaba.

**Table 1:** **Demographic details of the participants in each experiment**.

	*N*	Age	AOE	DOE	LQ
**Experiment I**	26	17 ± 0.3	12.6 ± 0.3	4.5 ± 0.2	81 ± 18
	(15 females)	(16−17)	(12−13)	(4.3−4.8)	(38−100)
**Experiment II**	24	17 ± 0.3	12.5 ± 0.3	4.5 ± 0.2	87 ± 13
	(18 females)	(16−17)	(12−13)	(4.3−4.8)	(63−100)

*The number of participants (N), age, age of first exposure (AOE) to English, duration of exposure (DOE) to English, and laterality quotient (LQ) are shown as the mean ± SD. Ranges are shown in brackets*.

#### Stimuli and Tasks

We visually presented stimuli, which were English sentences identical to those used after training sessions in our previous studies (Nauchi and Sakai, 2009; Sakai et al., 2009). In brief, we selected 42 high-frequency English verbs (20 transitive and 22 intransitive, including 12 unergatives and 10 unaccusatives (Yusa, 2003)), and used them to make sentence stimuli with other high-frequency words; each verb was used 1–3 times to test different properties of these verbs. Each stimulus consisted of a key sentence and its associated sentence, the latter of which was either syntactically normal or anomalous in the Syn task. It is generally hard for L2 learners to acquire correct argument structures and their associated syntactic knowledge. For instance, Japanese students learning English as L2 tend to omit an object and to accept such incorrect sentences (*) as “Do you often meet Mary?—*Yes, I often meet,” since objects of transitive verbs can be omitted quite freely in many languages including Japanese. In this way, our task properly tested the structural properties of verbs. The stimuli contained several other types of errors, such as an incorrect use of intransitive verbs (“*You can kill many monsters in the game.*—*You will die them in the game.”) or subject drop (“*John often comes to my house.*—*Soon will come.”), requiring the relational properties of the constituents. Because these ungrammatical sentences cannot be judged as incorrect by semantic information alone, the accuracy of the Syn task appropriately reflected individual syntactic abilities. In the Spe task, a typographical error was included to test the English orthography of words. Moreover, the sets of sentences were basically the same in the Syn and Spe tasks, and multiple words were naturally related to one another to understand the meanings of these sentences in both tasks.

All behavioral data were acquired outside the MR scanner. At the initiation of every trial of 7 s, a cue, indicating whether the task was Syn or Spe, was shown for 400 ms. Next, a set of sentence stimuli was shown for 6400 ms. The participants were instructed to read the sentences silently, and to indicate whether or not the sentences contained an error by pushing one of two buttons. For each task, *five trials* were consecutively performed as a task block for better concentration on each type of task, and these task blocks were alternately performed. The initial order of task blocks was counterbalanced across participants, thereby eliminating any effects of the order of presentation [i.e., Syn—Spe—Syn… or Spe—Syn—Spe…]. Participants performed 50 trials, and after a short break, they performed another 50 trials, accomplishing 100 trials in total (50 each for Syn and Spe; 50 each for correct and incorrect stimuli, completely randomized). As in the previous studies with the same tasks, trials with no response within the allowed time were included as incorrect ones in calculating the accuracy of each task; a response within 1.5 s was regarded as the response to the trial just one before. As regards RTs, only trials with correct responses were included. After the task instruction and brief explanation, the participants received 10 training trials (five for Syn, five for Spe). Additional 10 trials were provided for some participants who needed to adapt to the time constraint of the tasks.

To test general skills in word processing, a verbal fluency task in L1 (Japanese) was performed. For a given single letter in hiragana, participants were asked to write as many words (except proper nouns) starting from this letter as possible within 60 s. The verbal fluency was separately tested in three trials with different letters, which consisted of an easier letter (*sa*) and two harder letters (*ri* and *nu*).

#### MR Image Acquisition

The scans were conducted on a 3.0 T scanner (Signa HDxt; GE Healthcare, Milwaukee, WI, USA) with an 8-channel phased array head coil. We set the axial sections of the brain in parallel with its anterior to posterior commissure (AC-PC) line. We scanned 50 axial slices that were 3-mm thick without a gap, covering the whole brain, with a diffusion-weighted spin-echo echo-planar imaging sequence (*b*-value = 4000 s/mm^2^, repetition time (TR) = 17 s, echo time (TE) = 110 ms, field of view (FOV) = 192 × 192 mm^2^, resolution = 3 × 3 mm^2^, number of excitations = 1). For this scanning, a single image without diffusion-weighting (b0) was initially acquired, and then diffusion-weighting was isotropically distributed along 60 diffusion-encoding gradient directions. After completion of the diffusion MRI session, high-resolution T1-weighted images of the whole brain (192 axial slices, 1 × 1 × 1 mm^3^) were acquired from all participants with a fast spoiled gradient recalled acquisition in the steady state sequence (*TR* = 10 ms, *TE* = 4 ms, *FA* = 25°, FOV = 256 × 256 mm^2^).

#### Diffusion MRI Data Analyses

We used *q*-ball imaging (Tuch, 2004), a *q*-space analysis of diffusion MRI, and performed data analyses using FSL [Oxford Centre for Functional MRI of the Brain’s (FMRIB) Software Library 4.1.9^1^] and FMRIB’s Diffusion Toolbox (FDT) 2.0 (Smith et al., 2004). Diffusion-weighted images were first resliced to an isotropic voxel size of 1 mm^3^, and then eddy current distortions and motion artifacts were corrected using affine registration to the b0 image. We then extracted the brain shape from the b0 image, and created a binary mask image (zero for the outside of the brain) for each participant to calculate the diffusion tensor, three eigenvectors, and FA in each voxel inside the brain. Fiber orientations were estimated for the constant solid angle orientation distribution functions (ODFs) using the spherical harmonic decomposition and residual bootstrapping algorithm included in the QBOOT (*q*-ball ODFs and residual bootstrap) toolbox in FSL (Sotiropoulos et al., 2013).

We performed tractography by a single-seed approach in the native space using the probtrackx tool of FDT; we basically followed the procedures described previously (Ohta et al., 2013). Probabilistic fiber tracking was initiated from each voxel within a seed mask to generate 5000 streamline samples in any direction, with a step length of 0.5 mm, a maximum number of steps of 2000, a curvature threshold of 0.2 (±78.5°), and a loopcheck option. Each voxel value represented the total number of streamline samples passing through that voxel, making a connectivity distribution. The connectivity probability maps were then created by dividing the connectivity distribution with a waytotal value, i.e., the total number of generated streamline samples from the seed mask that had reached two waypoint masks. To remove any spurious connections, tracts were thresholded to include only voxels that had connectivity probability values of at least 0.2%. Thresholded tracts were then binarized using the fslmaths function of FSL. In order to prevent the misplacement of seed and waypoint masks, all tracts were inspected to confirm that they were not touching the peripheral edges of the masks.

#### Identification of the Arcuate and IFOF

**Figure 1:**
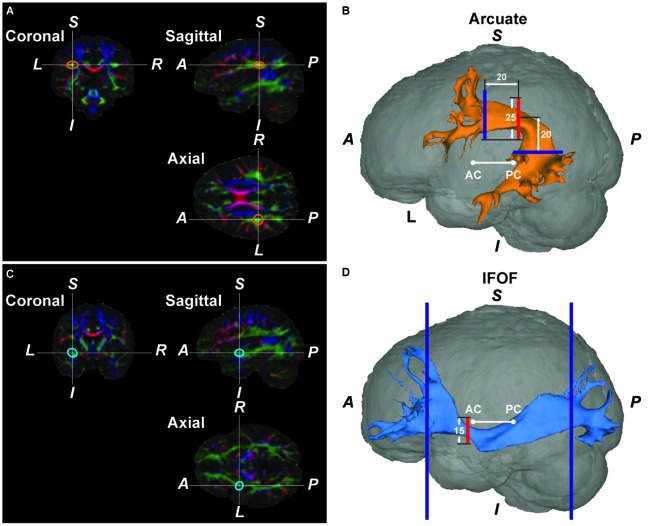
**The Arcuate and inferior fronto-occipital fasciculus (IFOF) reconstructed by diffusion magnetic resonance imaging (MRI) in a typical participant. (A)** A color-coded map of fiber bundles in the native space of a participant. In the Montreal Neurological Institute (MNI) space, we placed a “bottleneck mask” (a sphere of 10-mm radius) at a region where a narrower portion of the pathway was expected to pass. The mask was then transformed back to the native space. The orange ellipses denote the approximate outline of the transformed bottleneck mask on the left Arcuate. **(B)** The reconstructed left Arcuate. On a coronal slice, we generated a seed mask (shown in red), whose center was placed at the center of the transformed bottleneck mask on the left Arcuate. The waypoint masks are shown in blue. **(C)** A color-coded map of fiber bundles for the same participant. The blue ellipses denote the outline of the transformed bottleneck mask on the left IFOF. **(D)** The reconstructed left IFOF. On a coronal slice, we generated a seed mask (shown in red), whose center was placed at the center of the transformed bottleneck mask on the left IFOF. *A*, anterior; *P*, posterior;* S*, superior; *I*, inferior; *L*, left; *R*, right; AC, anterior commissure; PC, posterior commissure.

By using FMRIB’s Linear Image Registration Tool (FLIRT) on FSL, the b0 image was first coregistered to the individual T1-weighted image for each participant, and the T1-weighted image was spatially normalized to the Montreal Neurological Institute (MNI) space by using both affine and nonlinear transformation with FLIRT and FMRIB’s Nonlinear Image Registration Tool (FNIRT). To reliably set the *center* of a seed mask, we placed a “bottleneck mask” (a sphere of 10-mm radius) at a region (±35, −35, 30) in the MNI space where a narrower portion of the Arcuate was expected to pass, with reference to the atlases of white matter pathways (Catani and Thiebaut de Schotten, 2008; Oishi et al., 2011). With the transformation matrices and estimated deformation fields, the bottleneck mask was transformed back to the native space of each participant (orange ellipses in Figure 1A). On a participant’s color-coded map of fiber bundles, we confirmed that the center-of-gravity of the transformed bottleneck mask was included in the tract’s cross-section; the transformed point was manually adjusted to the center of the tract within a few millimeters [mean ± SD; Experiment: 3.8 ± 1.3 mm (left), 4.4 ± 2.1 mm (right); Experiment II: 3.8 ± 1.6 mm (left), 4.0 ± 1.1 mm (right)]. The amount of adjustments had no hemispheric difference according to the paired *t*-tests in Experiment I (*t*_(25)_ = 1.3, *P* > 0.1) or in Experiment II (*t*_(23)_ = 0.4, *P* > 0.6). On a coronal slice, we generated a *seed* mask of 25-mm square (the red square in Figure 1B), whose center was placed at the transformed point. The resultant seed mask included the entire cross-section of the Arcuate. These semi-automatic procedures allowed us to define seed masks in a consistent manner among participants.

To reliably extract the Arcuate alone, we used two *waypoint* masks of 30-mm squares (blue squares in Figure 1B) that were large enough to include the tract’s cross-sections. A coronal mask was placed 20-mm anterior to the seed mask in the native space, and an axial mask was placed 20-mm inferior to the seed mask’s center. For the coronal mask, its lowest edge was parallel to that of the seed mask at the same *z*-coordinates; both masks’ centers also had the same *x*-coordinates. The axial mask’s center had the same *x*-coordinates as the seed mask, and the *y*-coordinate of the axial mask’s center was initially set 10 mm posterior to the seed mask. By considering individual variability near the arching part of the pathways, we adjusted the *y*-coordinate within a few millimeters [Experiment I: 2.1 ± 2.7 mm (left), –1.3 ± 3.7 mm (right); Experiment II: 2.8 ± 2.5 mm (left), –1.2 ± 2.7 mm (right)]. The amount of adjustments had hemispheric differences in Experiment I (*t*_(25)_ = 5.1, *P* < 0.05) and in Experiment II (*t*_(23)_ = 7.3, *P* < 0.05), indicating that the curvature of the Arcuate was different in each hemisphere. We also used a set of exclusion masks in three planes to avoid the potential inclusion of fibers other than the Arcuate: a coronal mask (15-mm square) to exclude the IFOF, an axial mask (30-mm square) to exclude the corona-radiata, and a mid-sagittal plane to exclude commissural fibers. The coronal exclusion mask was the same as the seed mask for the IFOF (see below), and the axial exclusion mask was placed 10 mm superior to the center of the coronal exclusion mask, and individually adjusted in the axial plane to cover a narrower portion of the central vertical fibers on the color-coded map of fiber bundles.

As regards the IFOF, we again placed a bottleneck mask (a sphere of 10-mm radius) at a region (±35, 0, −5) in the MNI space where a narrower portion of the IFOF was expected to pass, with reference to the atlases of white matter pathways. The bottleneck mask was transformed back to the native space of each participant (blue ellipses in Figure 1C). We confirmed that the center-of-gravity of the transformed bottleneck mask was included in the tract’s cross-section; the transformed point was manually adjusted to the center of the tract within a few millimeters [Experiment I: 4.9 ± 1.3 mm (left), 5.1 ± 2.0 mm (right); Experiment II: 4.5 ± 1.1 mm (left), 4.7 ± 2.0 mm (right)]. The amount of adjustments had no hemispheric difference according to the paired *t*-tests in Experiment I (*t*_(25)_ = 0.5, *P* > 0.5) or in Experiment II (*t*_(23)_ = 0.3, *P* > 0.6). On a coronal slice, we generated a seed mask of 15-mm square (the red square in Figure 1D), whose center was placed at the transformed point. To include longer tracts connecting both the IFG and occipital regions, we used waypoint masks at two coronal planes in each individual hemisphere: one at the head of the caudate nucleus, and the other at the parieto-occipital sulcus (blue squares in Figure 1D; Forkel et al., 2014). We also used a set of exclusion masks in two planes to avoid the potential inclusion of fibers other than the IFOF: a coronal mask to exclude the Arcuate and superior fronto-occipital fasciculus (Forkel et al., 2014), and a mid-sagittal mask to exclude commissural fibers. The coronal exclusion mask covered only the upper half of the native MR image, and was placed at the anteroposterior midpoint of the image.

#### ROI Selection to Minimize Individual Variances in the Fiber Tract’s Thickness

**Figure 2:**
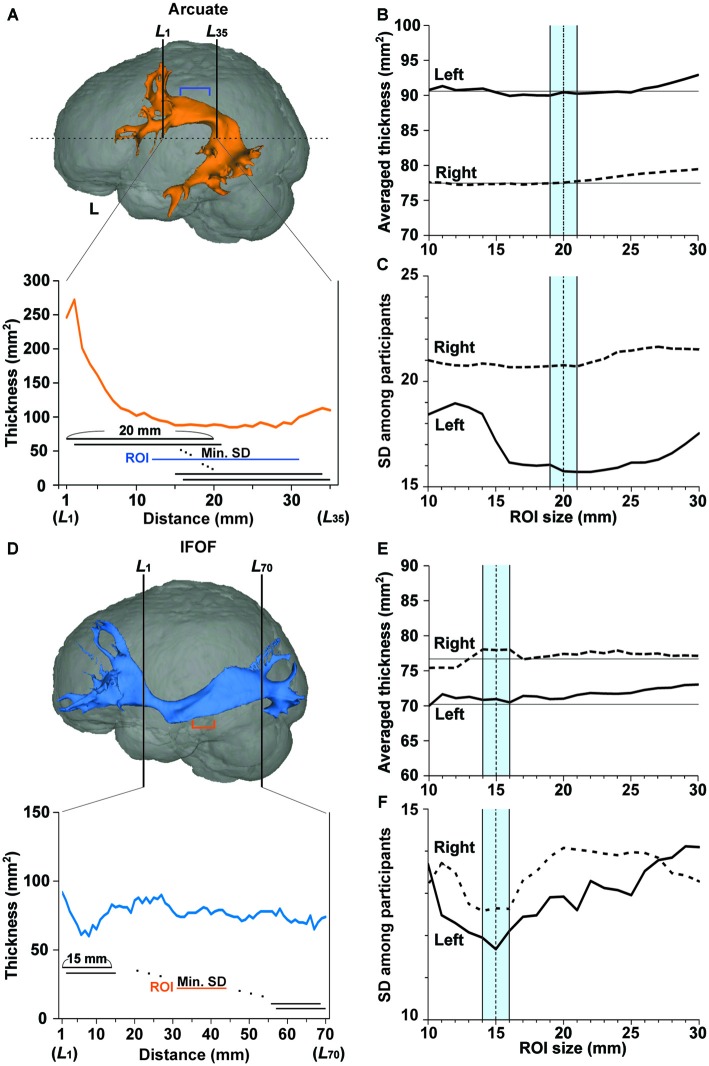
**Region of interest (ROI) selection to minimize individual variances in the fiber tract’s thickness. (A)** A ROI (a segment shown by blue lines) in the left Arcuate reconstructed for the participant shown in Figure 1. Thickness was defined as the number of voxels (voxel size, 1 mm^2^) at each coronal section of the tracked fibers. In the lower graph, the tract’s thickness is shown at each of the *L*_1_~*L*_35_ cross-sections for the upper half of the brain (above the horizontal dashed line). Within a tract segment of a fixed length (e.g., 20 mm), we calculated the standard deviation (SD) of the tract’s thickness, and slid the segment between *L*_1_ and *L*_35_. Among these segments, the segment with a minimal SD, i.e., with the most uniform thickness, was selected as a ROI. **(B,C)** The averaged thickness and SD for various ROI sizes in the Arcuate. The mean thickness in the ROI was further averaged, and the SD was obtained among the participants. The averaged thickness or SD in the left and right hemispheres is shown by the solid and dashed lines, respectively. To minimize the SD, we chose a ROI size in the range of 19–21 mm (blue-shaded), in proportion to the individual size of the brain. **(D)** A ROI (a segment shown by orange lines) in the left IFOF reconstructed for the same participant. In the lower graph, the tract’s thickness is shown at each of the *L*_1_~*L*_70_ cross-sections. Among these segments, the segment with a minimal SD was selected as a ROI. **(E,F)** The averaged thickness and SD for various ROI sizes in the IFOF. The averaged thickness or SD in the left and right hemispheres is shown by the solid and dashed lines, respectively. We chose a ROI size in the range of 14–16 mm (blue-shaded), in proportion to the individual size of the brain.

The *thickness* of the fiber tract was defined as the number of voxels (voxel size, 1 mm^3^) at a certain section of the tracked fibers. Here we chose coronal sections, because the portions without branching in the Arcuate or IFOF were nearly horizontal. We measured the thickness along the Arcuate in each hemisphere for the length of 35 mm, i.e., cross-sections* L*_1_~*L*_35_ of the upper half of the image, corresponding to the 94th~128th coronal slices from the anterior end of the image (see Figure 2A). Within a tract segment of a fixed length (e.g., 20 mm), we calculated the SD of the tract’s thickness, and slid the segment between *L*_1_ and *L*_35_. Among these segments, the segment with a minimal SD, i.e., with the most uniform thickness, was selected as a ROI. To objectively determine its appropriate length or *ROI size*, we varied it from 10 to 30 mm, and the “mean thickness” was calculated among the selected cross-sections. The mean thickness was further averaged among the participants (Figures 2B,C). The averaged thickness started to increase when the ROI sizes exceeded 20 mm in both hemispheres (Figure 2B), indicating the contribution from branching portions. In the left hemisphere, the SD of the mean thickness among participants was lowest for the ROI size of around 20 mm (Figure 2C). We thus chose a ROI size in the range of 19–21 mm, in proportion to the individual size of the brain. For 47 out of 52 tracts in all hemispheres tested, the final ROIs did not reach the end of *L*_1_ or *L*_35_; thus the search volume of *L*_1_~*L*_35_ was large enough to determine most ROIs.

As regards the IFOF, we measured thickness for the length of 70 mm, i.e., cross-sections* L*_1_~*L*_70_, corresponding to the 84th~153th coronal slices from the anterior end of the image (see Figure 2D). Within a tract segment of a fixed length (e.g., 15 mm), we calculated the SD of the tract’s thickness, and slid the segment between *L*_1_ and *L*_70_. Among these segments, the segment with a minimal SD was selected as a ROI. To objectively determine an appropriate length or ROI size, we varied it from 10 to 30 mm, and calculated the “mean thickness.” The averaged thickness among the participants started to increase when the ROI sizes exceeded 15 mm in the left hemisphere (Figure 2E). In both hemispheres, the SD of the mean thickness among participants was lowest for the ROI size of around 15 mm (Figure 2F). We thus chose a ROI size in the range of 14–16 mm, in proportion to the individual size of the brain. For all tracts tested, the final ROIs did not reach the end of *L*_1_ or *L*_70_.

#### FA Analyses

As described in the above sections, ROIs were determined on the basis of the connectivity probability and resultant thickness. After selecting ROIs, voxels with FA values of 0.2 or higher were selected, and this thresholding was used only in FA analyses. The thresholding has been widely used in previous studies to avoid the uncertainty of anisotropy measurements in the peripheral regions of fibers. We calculated the mean FA within the ROI, and examined the relationships between the accuracy of the Syn task and FA in each pathway after removing the effects of the accuracy of Spe, gender, and LQ, using the ppcor (partial and semi-partial correlation) package^2^ in R software^3^.

### Experiment II

In Experiment II, only monozygotic twin pairs (24 twins) were included (Table 1); we set the first three inclusion criteria (i)–(iii) used in Experiment I. Note that the criterion for the accuracy of Spe was *not* employed in Experiment II. Eight participants were analyzed in both Experiment I and II, but these analyses were independent and did not constitute selection and selective evaluation analyses. As in Experiment I, the participants performed the Syn, Spe, and verbal fluency tasks, and MR imaging acquisition was conducted with the same parameters. The Arcuate and IFOF were identified in each individual hemisphere, as described above. We examined correlations within twin pairs separately for the behavioral data and the structural (FA/thickness) data. To examine the difference between correlations, we statistically compared correlation coefficients, using the cocor (comparing correlations) package^4^ in R software (Diedenhofen and Musch, 2015).

### Effects of Different Imaging Parameters on FA

In a pilot experiment for 13 participants (including four pairs of monozygotic twins) from Experiment I/II, diffusion MRI data were acquired with a *b*-value of 1000 s/mm^2^ (TR = 17 s, TE = 90 ms, FOV = 256 × 256 mm^2^, resolution = 2 × 2 mm^2^, number of excitations = 1), in addition to diffusion MR data with a *b*-value of 4000 s/mm^2^ (see “MR Image Acquisition” Section). We scanned 67 axial slices that were 2-mm thick without a gap, covering the whole brain. A single b0 image was initially acquired, and then diffusion-weighting was isotropically distributed along 60 diffusion-encoding gradient directions, as in Experiment I/II. In our MRI system, we were able to set a smaller voxel size for a lower *b*-value of 1000 s/mm^2^ (2 mm^3^) than that for a *b*-value of 4000 s/mm^2^ (3 mm^3^). We confirmed that FA from the imaging with a *b*-value of 4000 s/mm^2^, which has been used for improved reconstruction of the fiber tracts (see “Introduction” Section), was consistent with FA obtained with a lower *b*-value and higher resolution. By using FDT 3.0 and FLIRT on FSL 5.0.4, the individual b0 image with the *b*-value of 1000 s/mm^2^ was coregistered to that with the *b-value* of 4000 s/mm^2^. With the same transformation matrices, an individual FA map with a *b*-value of 1000 s/mm^2^ was also spatially transformed for each participant. We then compared the FA values in the left Arcuate obtained with different *b*-values, within the tract reconstructed by *q*-ball imaging with the *b*-value of 4000 s/mm^2^.

## Results

### Experiment I

#### Behavioral Data

Behavioral data of the Syn and Spe tasks are shown in Table 2. For the verbal fluency task, we evaluated each individual’s performance by adding together the number of words produced for all trials (mean ± SD: 26.2 ± 6.3). The performance of the verbal fluency task was not correlated with any of the accuracy/RTs in Syn or Spe (*P* > 0.2). The accuracy of Syn was significantly lower than that of Spe (*t*_(25)_ = 11.4, *P* < 0.0001), and the RTs of Syn were significantly longer than those of Spe (*t*_(25)_ = 12.6, *P* < 0.0001), indicating that the Syn task was more demanding than the Spe task.

**Table 2:** **Behavioral data of the participants**.

	Accuracy (%)		RTs (ms)	
	Syn	Spe	Syn	Spe
**Experiment I**	61 ± 11	86 ± 4.9	4778 ± 383	4324 ± 410
	(42−88)	(80−100)	(3989−5378)	(3503−5006)
**Experiment II**	60 ± 8	82 ± 9.2	4806 ± 489	4316 ± 529
	(44−73)	(68−100)	(3989−5877)	(3503−5587)

*Only trials with correct responses were included for the reaction times (RTs). Data are shown as the mean ± SD. Ranges are shown in brackets*.

**Figure 3:**
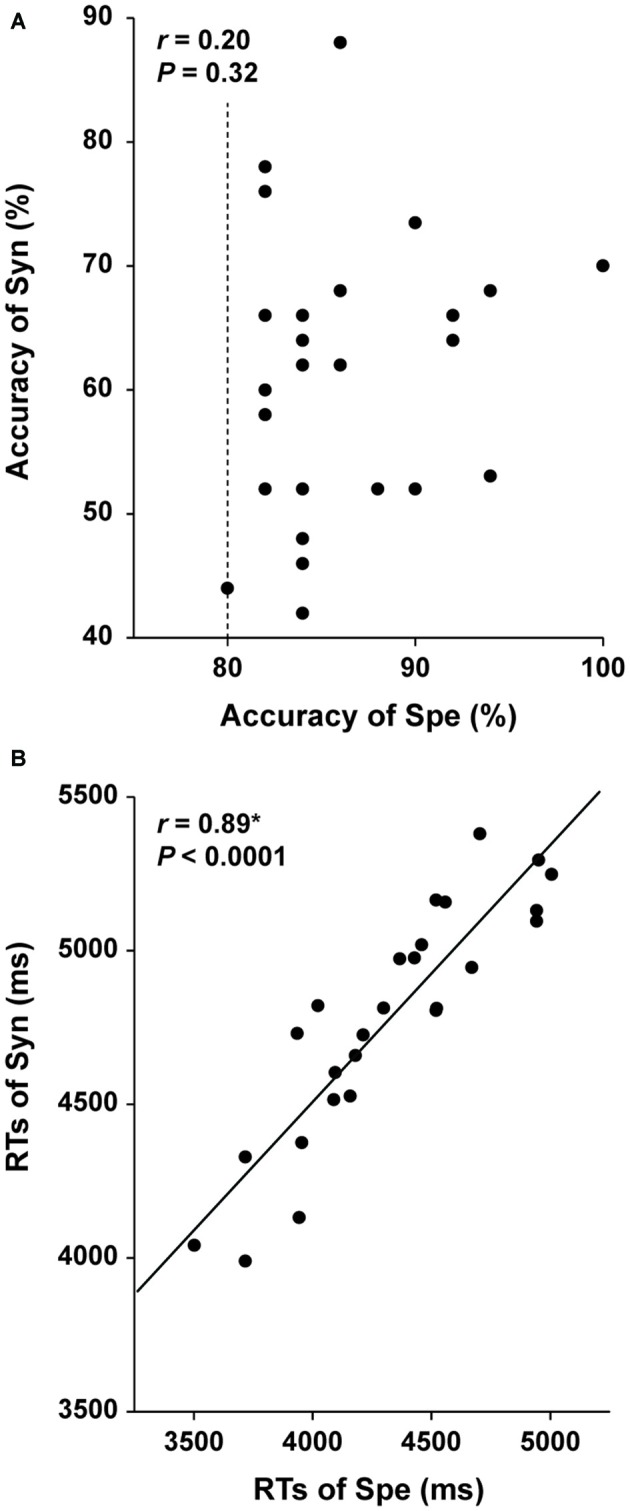
**Independence of the accuracy of the Syn and Spe tasks.** Individual behavioral parameters in Experiment I are plotted for comparing the two tasks. The dotted line indicates an inclusion criterion of Spe employed in Experiment I. The accuracy of Syn and Spe showed no significant correlation **(A)**, while reaction times (RTs) of Syn and Spe were highly correlated **(B)**. An asterisk at *r* indicates corrected *P* < 0.05.

If a behavioral parameter reflected general cognitive factors, such as task difficulty and reading proficiency, which were commonly involved in the Syn and Spe tasks with some differences in degree, then that parameter should show a correlation between Syn and Spe among the participants, when considered their individual differences regarding these factors. For example, a slower reader would show longer RTs for both Syn and Spe. Therefore, as a contraposition, if another behavioral parameter did not show such a correlation between Syn and Spe among the participants, that parameter reflects at least distinct factors required in each task. We thus examined the relationships between the performances of the two tasks. The accuracy of Syn and that of Spe did not show a significant correlation (*r* = 0.20, *P* = 0.32; Figure 3A), suggesting that the accuracy mainly reflected distinct abilities required in each task. On the other hand, the RTs of Syn and Spe showed a highly positive correlation (*r* = 0.89, *P* < 0.0001; Figure 3B), indicating that the RTs reflected general cognitive factors common to both tasks. We observed that the correlation coefficient between RTs was significantly larger than that between the accuracy (*Z* = 3.1, *P* < 0.05) by using the cocor (comparing correlations) package in R software, confirming the different natures of the accuracy and RTs. These results indicate that the *accuracy* of each task for the participants reflected individual abilities required in each task.

#### Structural Properties of the Arcuate and IFOF

Structural properties obtained in each ROI of the Arcuate and IFOF are shown in Table 3. Within the tested group, the distribution was not significantly different from the normal distribution for the mean FA and thickness in each ROI of the four pathways [the Shapiro and Wilk (1965) test; *P* > 0.08]. As regards the mean FA, a two-way repeated-measures analysis of variance (rANOVA) [pathway (Arcuate, IFOF) × hemisphere (left, right)] showed a significant main effect of pathway (*F*_(1,25)_ = 54.2, *P* < 0.001), but with neither a main effect of hemisphere (*F*_(1,25)_ = 0.86, *P* = 0.36) nor an interaction (*F*_(1,25)_ = 0.73, *P* = 0.4). According to a paired *t*-test (two-tailed) in each hemisphere, the mean FA in the ROI of the IFOF was significantly higher than that of the Arcuate (left: *t*_(25)_ = 5.3, *P* < 0.001; right: *t*_(25)_ = 5.0, *P* < 0.001). With respect to the mean thickness, an rANOVA showed a significant main effect of pathway (*F*_(1,25)_ = 9.0, *P* < 0.01) and an interaction (*F*_(1,25)_ = 26.9, *P* < 0.001), but no main effect of hemisphere (*F*_(1,25)_ = 1.2, *P* = 0.3). Paired *t*-tests showed that the mean thickness in the ROI of the left Arcuate was significantly larger than that of the right Arcuate (*t*_(25)_ = 3.7, *P* < 0.005), while that of the right IFOF was marginally larger than that of the left IFOF (*t*_(25)_ = 2.1, *P* = 0.049; significance level at *α* = 0.025, Bonferroni-corrected). These results indicated that the Arcuate and IFOF were different in terms of both macroscopic and microscopic properties, i.e., thickness and FA, respectively, even when the same objective methods for ROI selection were employed.

**Table 3:** **Structural properties of the Arcuate and IFOF**.

	Mean FA		Mean thickness (mm^2^)		
	Left	Right	Left	Right	Difference
Arcuate	0.39 ± 0.02	0.39 ± 0.02	90 ± 16	78 ± 21	*P* < 0.005
IFOF	0.44 ± 0.04	0.43 ± 0.04	71 ± 11	78 ± 13	*P* = 0.049

*For each pathway of either hemisphere, the mean FA or mean thickness in each ROI was obtained in Experiment I. Data are shown as the mean ± SD among the participants (N = 26). Hemispheric difference was assessed by paired t-tests*.

#### FA of the Left Arcuate as an Indicator of Individual Performances for the Syn Task

**Figure 4:**
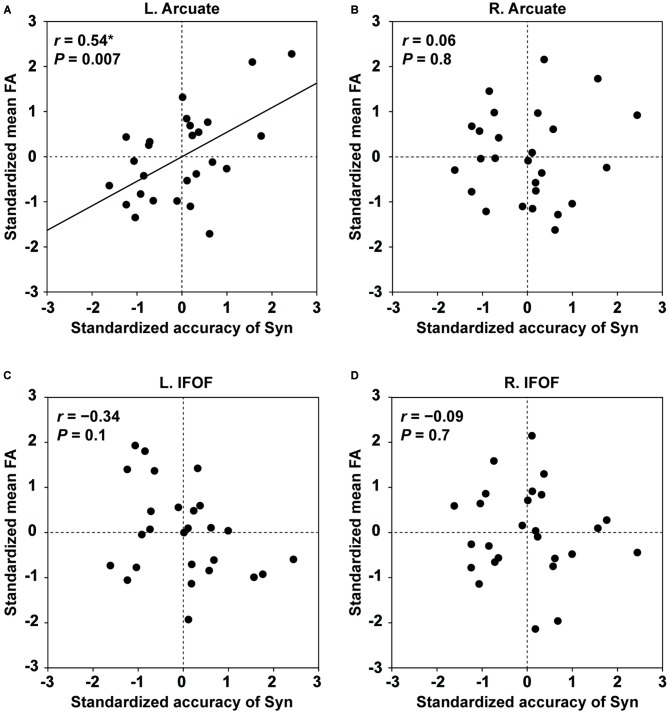
**Fractional anisotropy (FA) of the left Arcuate as an indicator of individual performances for the Syn task.** The scatter plots of the standardized accuracy of Syn and the standardized mean FA in each ROI are shown for the left Arcuate **(A)**, right Arcuate **(B)**, left IFOF **(C)**, and right IFOF **(D)**. The effects of the standardized accuracy of Spe, gender, and laterality quotient (LQ) were removed. Uncorrected *P* values are also shown for each panel. Only the left Arcuate showed a significant correlation between FA and the accuracy of Syn.

Within the tested group, the distribution was not significantly different from the normal distribution for the behavioral performances (the accuracy of Syn, and the number of produced words in the verbal fluency task), as well as for the mean FA in each ROI of the four pathways (the Shapiro-Wilk test; *P* > 0.1). For each of the four pathways, we performed partial correlation analyses between the standardized mean FA and the standardized accuracy of Syn (two-tailed, significance level at *α* = 0.0125, Bonferroni-corrected), removing the effects of other major independent factors: the standardized accuracy of Spe, gender, and LQ (there were no correlations among these factors: *r* < 0.3, *P* > 0.3). The mean FA of the left Arcuate showed a significant positive correlation with the accuracy of Syn (*r* = 0.54, *P* = 0.007; Figure 4A). In contrast, the mean FA in the ROI of the right Arcuate was not significantly correlated with the accuracy of Syn (*r* = 0.06, *P* = 0.8; Figure 4B). As regards the IFOF, the mean FA in either hemisphere was not significantly correlated with the accuracy of Syn (left: *r* = −0.34, *P* = 0.1; right: *r* = −0.09, *P* = 0.7; Figures 4C,D). Additionally, considering that FA (≥0.2) in each ROI might not be normally distributed, we tested all analyses above for the median FA instead of the mean, and replicated the overall results (a partial correlation between the median FA in the left Arcuate and the accuracy of Syn; *r* = 0.56, *P* = 0.003). Regarding the accuracy of Spe, we performed partial correlation analyses between the standardized mean FA and the standardized accuracy of Spe, thereby removing the effects of the standardized accuracy of Syn, gender, and LQ. The mean FA of the left Arcuate did not show a significant correlation with the accuracy of Spe (*r* = 0.04, *P* = 0.9). None of the other pathways showed a significant correlation with the accuracy of Spe (*P* > 0.19). Note that the range of the accuracy of Spe was limited due to the fifth inclusion criterion (see “Participants” Section), and that the statistical power was lower. To further confirm the absence of the effects of the load associated with word processing, we also performed partial correlation analyses between the standardized mean FA and the standardized performance of the verbal fluency task. The mean FA of the left Arcuate was not correlated with the performance of the verbal fluency task (*r* = 0.13, *P* = 0.5), and the other pathways did not show significant correlations (*P* > 0.5). These results demonstrate that FA of the left Arcuate can be a selective indicator of individual performances for the Syn task.

### Experiment II

#### Behavioral Correlations within Twin Pairs

**Figure 5:**
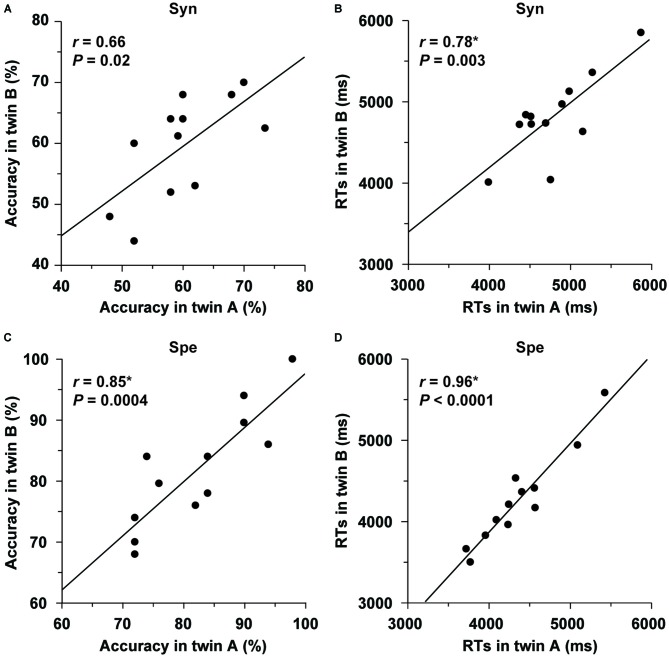
**Behavioral correlations within twin pairs. (A–D)** Correlations for accuracy/RTs of the Syn and Spe tasks are shown separately, and plotted for each pair of monozygotic twins. Each twin was randomly assigned as either twin A or twin B in a pair. The twin pairs showed significant correlations for task performances.

Behavioral data for monozygotic twins are shown in Table 2. Each monozygotic twin was randomly assigned as either twin A or twin B in a pair. Within the twin A or twin B group, the distribution of each of the task performances in L2 was not significantly different from the normal distribution (the Shapiro-Wilk test; *P* > 0.1). We examined correlations within twin pairs for the behavioral data (two-tailed, significance level at *α* = 0.0125, Bonferroni-corrected). As regards the Syn task, the correlation of the accuracy within pairs was marginally correlated (*r* = 0.66, *P* = 0.02; Figure 5A), while the RTs were significantly correlated (*r* = 0.78, *P* = 0.003; Figure 5B). As regards the Spe task, the twins exhibited highly correlated performances in terms of accuracy (*r* = 0.85, *P* = 0.0004; Figure 5C) and RTs (*r* = 0.96, *P* < 0.0001; Figure 5D). Additionally, the correlation coefficient for the RTs of Spe was significantly larger than that for the accuracy of Syn (*P* < 0.05). With respect to the verbal fluency task, which was also normally distributed (*P* > 0.09), the total number of produced words was significantly correlated for the twins (*r* = 0.81, *P* = 0.001).

To further confirm that these correlations did not occur by chance, each twin was randomly assigned to a new pair to produce 10,000 permutations (Sakai et al., 2004). These random pairings resulted in a normal distribution (RTs of Syn: mean *r* = −0.07, *SD* = 0.27; accuracy of Spe: mean *r* = −0.08, *SD* = 0.29; RTs of Spe: mean *r* = −0.08, *SD* = 0.26; verbal fluency: mean *r* = −0.08, *SD* = 0.29), establishing that the above results were actually due to factors that each twin pair had in common.

#### Structural Correlations within Twin Pairs

**Figure 6:**
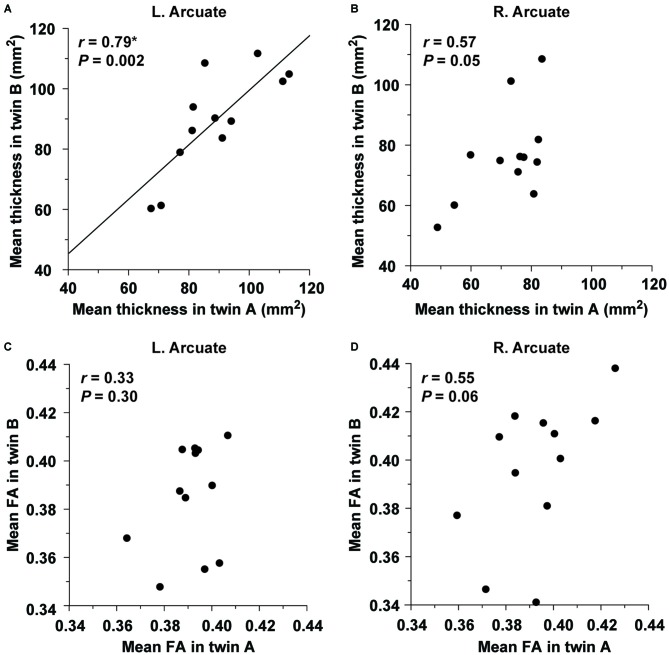
**The structural correlation of the left Arcuate within twin pairs.** The mean thickness in the ROI of the left **(A)** or right **(B)** Arcuate is plotted for each pair of monozygotic twins. The plot of the mean FA in the ROI of the left **(C)** or right **(D)** Arcuate is also shown. Note the significant correlation for the mean thickness of the left Arcuate alone.

Within the twin A or twin B group, the distribution of thickness/FA in the ROIs of the Arcuate and IFOF was not significantly different from the normal distribution (the Shapiro-Wilk test; *P* ≥ 0.05). We analyzed the structural correlations within twin pairs for the Arcuate (two-tailed, significance level at *α* = 0.0125, Bonferroni-corrected). As regards the mean thickness, the twins were highly correlated in the left hemisphere (*r* = 0.79, *P* = 0.002; Figure 6A), but not in the right hemisphere (*r* = 0.57, *P* = 0.05; Figure 6B). Random pairings for the thickness of the left Arcuate resulted in a normal distribution (mean *r* = −0.05, *SD* = 0.29), confirming that the significant correlation was due to factors that each twin pair had in common. With respect to the mean FA, the correlation was not significant in either hemisphere (left: *r* = 0.33, *P* = 0.30; right: *r* = 0.55, *P* = 0.06; Figures 6C,D). For the IFOF, the correlation was not significant in either hemisphere as regards the mean thickness (left: *r* = 0.19, *P* = 0.56; right: *r* = 0.05, *P* = 0.89) or the mean FA (left: *r* = 0.57, *P* = 0.05; right: *r* = −0.21, *P* = 0.50). Indeed, the correlation coefficient for the thickness of the left Arcuate was significantly larger than that of the left IFOF (*P* < 0.05).

In Experiment II, where the criterion for the accuracy of Spe was not employed, the accuracy of Syn and that of Spe were correlated (*r* = 0.56, *P* < 0.01). Because the assumption of independence between the accuracy of the two tasks in Experiment I did not apply in Experiment II, we considered that further correlation analyses among multiple metrics/ properties of behavior and structure (e.g., cross-correlation analyses between the accuracy of Syn in twin A and structural properties in twin B, and vice versa) were inconclusive in Experiment II.

### Effects of the Imaging Parameters on FA

**Figure 7:**
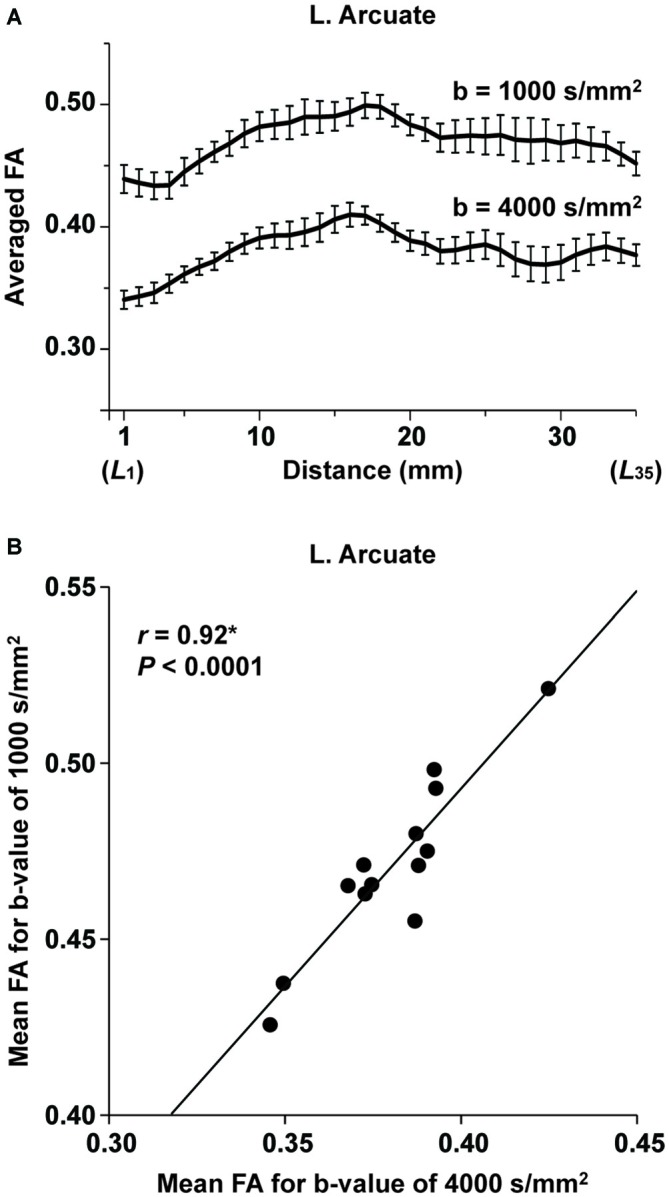
**The consistency of FA for different imaging parameters. (A)** At each of the *L*_1_~*L*_35_ cross-sections along the left Arcuate (see Figure 2A), FA with a *b*-value of 4000 s/mm^2^ (voxel size: 3 mm^3^) or 1000 s/mm^2^ (voxel size: 2 mm^3^) was further averaged among 13 participants (error bars, standard errors of the mean). **(B)** A correlation between FA for different *b*-values. The mean FA among the voxels within *L*_1_~*L*_35_ is plotted for each participant.

In the pilot experiment, we examined whether the FA values obtained with different *b*-values were correlated. As shown in Figure 7A, the overall profiles of FA averaged among the participants were similar for both *b*-values throughout the range of *L*_1_~*L*_35_ in the left Arcuate, although FA itself was higher for 1000 s/mm^2^. To examine the global tendency, we calculated the mean FA among the voxels in the entire range of* L*_1_~*L*_35_ for each participant, and examined the correlation between the mean FA values at different *b*-values. As shown in Figure 7B, the mean FA with the higher *b*-value was exactly correlated with that having a lower *b*-value (*r* = 0.92, *P* < 0.0001). These results confirm that the fiber tracts with a *b*-value of 4000 s/mm^2^ faithfully replicated the properties of those with a lower *b*-value.

## Discussion

The main results are summarized as follows. First, we improved the seed definition in a semi-automatic manner to reliably track the Arcuate and IFOF (Figure 1), and successfully identified those fiber tracts in both hemispheres of all participants. We then objectively selected their ROIs, thereby minimizing the variances in thickness among participants (Figure 2), and further clarified that the Arcuate was significantly thicker in the left hemisphere than in the right, while the IFOF was marginally thicker in the right hemisphere (Table 3). Secondly, we revealed that the mean FA in the ROI of the left Arcuate was significantly correlated with the accuracy of the Syn task (Figure 4A). This positive correlation was independent of gender, handedness (LQ), and the orthographic knowledge while reading sentences (the accuracy of the Spe task), as well as of age, AOE, and DOE, which were already controlled among the participants (Table 1). Moreover, FA in the left Arcuate was not significantly correlated with the performance of the verbal fluency task. Because the accuracy of Syn was independent of that of Spe (Figure 3A), the accuracy of Syn mostly represented individual syntactic abilities in L2. Thirdly, within monozygotic twin pairs, significant correlations were observed in the RTs of Syn/Spe, the accuracy of Spe, and the verbal fluency in L1, while the accuracy of Syn was marginally correlated (Figure 5). Indeed, the correlation coefficient for the RTs of Spe was significantly larger than that for the accuracy of Syn. Finally, the mean thickness in the ROI of the left Arcuate was highly correlated within twin pairs (Figure 6A). The correlation coefficient for the thickness of the left Arcuate was significantly larger than that of the left IFOF. These results suggest that general task performances, as well as the thickness of the left Arcuate, are more associated with the shared genetic/environmental factors, whereas both of mutually correlated FA in the left Arcuate and individual syntactic abilities in L2 may be less prone to these shared factors.

There have been a few previous studies in which a ROI was selected to exclude branching or curved portions of the Arcuate. In a tractography study, a ROI of a 7-mm-long segment was chosen at a tightly bundled portion in each of the Arcuate and SLF, with some overlaps between the resultant ROIs for the two bundles (Tsang et al., 2009); those ROIs were defined in reference to the individual central sulcus, and the mean FA was obtained within each ROI. In another study, a ROI of a 10-mm-long segment was chosen in the Arcuate, where the averaged FA among participants was highest in the entire tract, instead of a curved region with lower FA (Yeatman et al., 2011). As a result of these selection procedures, the selected ROIs had similar positions in those studies, but some participants might have had lower FA with the ROIs. Moreover, it was not clear whether the ROIs were sufficiently large to represent the tract itself. Because our ROIs with uniform thickness were independent of FA, the present results would be free from any sampling bias of selecting particular regions with higher FA. In the present study, the mean FA in the ROI of the IFOF was significantly higher than that of the Arcuate in both hemispheres. The ROIs of the IFOF did not include the thinnest portion that was previously found to have a lower and more unstable FA (Yeatman et al., 2012), probably due to other intermixed tissue; such portions should be avoided in examining the relationship between FA and behavioral measurements. While FA did not show a hemispheric difference for the Arcuate or IFOF in the present study, we found that the left Arcuate was clearly thicker than the right, a finding which may be related to the left-lateralized language functions. The IFOF, on the other hand, was marginally thicker in the right hemisphere. This opposing lateralization of the Arcuate and IFOF is consistent with a previous study, where the laterality was assessed by the number of streamlines in tractography (Thiebaut de Schotten et al., 2011). The thickness in our ROIs was similar to the relative number of streamlines of each pathway, while successfully excluding the portions of branching or contamination with other tissue. Our semi-automatic methods of seed definition and ROI selection would be applicable to other pathways as well, and would thus be of use for the investigation of hemispheric or individual differences.

There were some limitations in the present methods. We minimized subjective procedures in seed and waypoint selection, but large individual variability hampered the totally automatic selection, and therefore slight manual adjustments were still necessary. This might have affected the results especially of the right Arcuate, which is known to be highly variable among individuals. However, the degree of adjustment was small in both hemispheres (mean, ≤5 mm) compared to the mask sizes (15–30 mm), and all tracts were inspected to confirm that they were not touching the peripheral edges of the masks (see “Materials and Methods” Section). Another potential concern was the thresholding used in the probabilistic tracking, in that there is no gold standard for the thresholding value. For connectivity probability values in each voxel, we used a threshold of 0.2% against individual waytotal values (i.e., values of the number of total *successful* streamlines; Galantucci et al., 2011; Ohta et al., 2013). Some studies used a fixed percentage [e.g., 0.001% in Griffiths et al. (2013)] against the number of all *generated* streamlines, which differed individually due to the different seed size for each participant. Given that most of the generated streamlines are rejected when reaching exclusion masks, or when going away from waypoint masks, the thresholding with successful streamlines would be more reliable. For determining an appropriate thresholding value, it is also important to consider the decrease in connectivity probability with distance from the seed mask (Morris et al., 2008). Such distance effects, as well as the concern that participants with lower waytotal values might result in a very low threshold, should be properly handled in future studies. Advances in thresholding would also contribute to examine the individual branching patterns, and to identify the anterior target regions, which might explain individual language abilities. The ROIs in the present study may depend on the gross anatomy of each tract, and further research using appropriate methods for quantifying individual variabilities in such features as branching patterns and curvature will be needed to understand the functions of language-related pathways.

Our previous studies using the same Syn and Spe tasks in L2 have shown that the activations in the dorsal F3t of the left IFG, as well as the leftward lateralization of this region’s volume, were correlated with the accuracy of the Syn task (Nauchi and Sakai, 2009; Sakai et al., 2009). We have also shown that the left IFG is specialized in syntactic processing in both L1 and L2 (Sakai et al., 2004, 2009; Tatsuno and Sakai, 2005). In the Syn-Spe contrast, the left middle temporal gyrus and angular gyrus were also significantly activated (Sakai et al., 2009). In the present study, we further showed that the structural property of the left Arcuate connecting the IFG and parietotemporal regions was correlated with the accuracy of the Syn task. The correlation coefficient between the RTs of Syn and Spe was significantly larger than that between the accuracy of the two tasks, indicating that the accuracy reflected distinct abilities required in each task, while the RTs reflected general cognitive factors, such as task difficulty and reading proficiency, common to both tasks. Moreover, short-term memory was not required in the present tasks. Although it is generally difficult to exclude the effects of semantic knowledge from any tasks using natural sentences, the ungrammatical sentences cannot be judged as incorrect by semantic information alone (see “Stimuli and Tasks” Section). Other remaining factors were further excluded from our partial correlation analyses between the accuracy of Syn and FA, by controlling the accuracy of Spe, because sentence reading was common to both tasks. Regarding the relationships between general cognitive abilities and L2 abilities, a previous study has reported that L2 reading proficiency was related to performances in a paired-associate test for unrelated word pairs and a phonological short-term memory task; this study used a general proficiency scale for L2 (The Interagency Language Roundtable scale; Linck et al., 2013). Future large-scale studies using a variety of behavioral measures are necessary to reveal which network is specifically involved in syntactic, semantic, and other processes supporting language functions.

With respect to the connectivity of the dorsal pathway, some studies have indicated that the fronto-temporal connection of the left Arcuate is further separable into two parallel pathways—one terminating in the posterior superior temporal gyrus, and the other terminating in the middle temporal gyrus (Glasser and Rilling, 2008)—while other studies have suggested that there are three segments in the Arcuate/SLF: a *long* segment between the frontal and the superior/middle temporal regions, a *posterior* segment between the inferior parietal and temporal regions, and an *anterior* segment/SLF between the frontal and inferior parietal regions (Catani et al., 2012). Moreover, the SLF may be further divided into three branches (Thiebaut de Schotten et al., 2012), whose correspondence to the anterior segment/SLF remains unclear. In regard to the ventral pathway, it includes the IFOF and uncinate fasciculus (UF) connecting the ventral frontal regions. The IFOF connects the ventral IFG (BA 45/47) and some posterior regions in the parietal, posterior temporal, and occipital cortex (Martino et al., 2010), whereas the UF connects the orbitofrontal cortex (BA 10/11/47) and anterior temporal lobe, including the temporal pole (Catani et al., 2012). The ventral pathway may also include partial connections, such as the EmC, external capsule (EC), middle longitudinal fasciculus (MdLF), and inferior longitudinal fasciculus (ILF; Makris and Pandya, 2009; de Champfleur et al., 2013), although their structural complexity, as well as the available scanning resolution, hampers the dissociation of these connections. There is thus need of a method to clarify the detailed connectivity of these pathways. One critical method would be to define seeds reliably and objectively, thereby combining the results from various imaging techniques, including diffusion MRI, functional MRI, and VBM.

The functional roles of the dorsal and ventral pathways have also been a matter of debate. A previous patient study reported that reduced FA in the left Arcuate was related to the degree of syntactic deficits in patients who suffered from primary progressive aphasia with some variants (Wilson et al., 2011), while other studies have claimed other functional roles, such as phonemic processing, lexico-semantic processing, or word learning (Glasser and Rilling, 2008; López-Barroso et al., 2013). As regards the ventral pathway, a previous functional/diffusion MRI study has suggested that the left ventral pathway, including the EmC, MdLF and ILF, is the sound-to-meaning mapping stream, focusing on regions activated for the contrast of meaningful speech vs. pseudospeech (Saur et al., 2008), and we have previously suggested that the left ventral IFG is involved in the selection and integration of semantic information (Homae et al., 2002). In our recent study of both normal and agrammatic patients, we demonstrated the existence of three syntax-related networks (Kinno et al., 2014): Network I includes the left dorsal IFG and a portion of the right dorsal pathway, and subserves syntax and its supportive system, and Network II includes a portion of the left dorsal pathway, working as a syntax and input/output interface, while Network III includes a portion of the left ventral pathway, and is related to the syntax-semantic interaction. As demonstrated by the present study, the left Arcuate, i.e., the left dorsal pathway of Network II, has more functional significance among these syntax-related pathways, in that this pathway’s FA reflected the individual syntactic abilities in L2. The left dorsal pathway would probably be involved in both bottom-up and top-down processing between lexico-semantic information and syntactic computation, consistent with previously proposed models (Hagoort, 2005; Friederici, 2012).

Several models for the functions of dorsal and ventral language pathways have been proposed. For instance, Friederici and others have proposed that the ventral pathways support syntactic phrase structure building, while the dorsal pathways, especially the one connecting the BA 44 and temporal regions, support the processing of syntactically complex sentences (Friederici, 2012). Hagoort and others have proposed another model, in which two crucial components of language systems, i.e., Memory (the mental lexicon) and Unification (the on-line assembly of lexical elements into larger structures at syntactic, semantic, and phonological levels), are represented in the temporal/inferior parietal regions and the left IFG, respectively, connected by the dorsal and ventral pathways (Hagoort, 2014). Bornkessel-Schlesewsky et al. (2015) proposed that the ventral pathways are engaged in time-independent auditory object recognition and combination, whereas the dorsal pathways are engaged in time-dependent sequence processing. Our results support the critical role of the dorsal pathway in the light of processing argument structures of verbs in L2 acquisition. Further research with standardized tests for performance in English as a foreign language that additionally cover other domains of syntactic ability is needed to reveal how various subcomponents of syntactic processing are subserved by the dorsal and ventral pathways. Any approaches to combine the anatomical significance of these dorsal/ventral pathways with findings from developmental and lesion studies would be useful in elucidating how particular pathways contribute to subprocesses in language functions.

According to a previous study on 9-year-old monozygotic and dizygotic twins, the contribution of non-shared environmental factors to individual variations of FA, which was averaged in the entire left SLF (probably extended to the Arcuate), was approximately 65%, while the contributions of genetic and shared environmental factors were 30% and 5%, respectively (Brouwer et al., 2010). Consistent with such contribution of non-shared environmental factors, the local FA can increase after several months of skill or vocabulary training even in adults (Scholz et al., 2009; Hosoda et al., 2013). On the other hand, it has been reported that the white matter volume was more dependent on genetic factors; the genetic contribution to the whole white matter volume was 85%, while the contribution of genetic factors to its FA was as low as 25% (Brouwer et al., 2012). There are thus at least three independent issues that should be clarified for twin studies in neuroscience: (1) shared vs. non-shared genetic/environmental factors; (2) local vs. whole brain; and (3) gray vs. white matter. In the present study, we showed that the mean thickness in the ROI of the left Arcuate was highly correlated within monozygotic twin pairs, while its FA was not. A previous MRI study on the gray matter of twins showed stronger shared genetic influences on the density (the proportion of gray matter) of the left IFG, which were more prominent than the genetic influences on the right IFG density (Thompson et al., 2001). Although twin studies on L2 acquisition are limited, a recent study with twins aged 14 has reported that 25% of the variance of L2 was due to non-shared environmental factors, where their L2 performances were evaluated by the UK National Curriculum rating system (Dale et al., 2012). Here we report that the effects of shared factors depended differentially on the L2 tasks, the accuracy, and RTs. More specifically, the correlations within twin pairs were strikingly high for RTs of both Syn and Spe, which reflected the speed of word recognition, reading, and/or decision making. Moreover, the high correlations for the accuracy/RTs of Spe, as well as those for verbal fluency in L1, suggest that such word-level knowledge is also strongly associated with shared genetic/environmental factors, while the marginal correlation for the accuracy of Syn suggests that syntactic abilities are less prone to such shared factors.

A recent diffusion MRI study has reported that FA varies along a tract (Johnson et al., 2014). Another study dissected the Arcuate into three segments (long, anterior, and posterior segments), and reported that genetic effects on FA were high for the left long, right anterior, and left posterior segments (Budisavljevic et al., 2015). Our present study employed semi-automatic analyses for the diffusion MRI data, thereby examining FA in consistent portions of the tracts, which were not possible in previous studies using the mean FA averaged in the entire tracts. Although the sample size of twin pairs was too small to draw a stronger conclusion, elaborate studies like the present study would indicate future directions for large-scale studies. Large-scale projects with hundreds of twins, such as Enhancing Neuroimaging Genetics through Meta-Analysis (ENIGMA) and Human Connectome Project (HCP), have reported that FA of major tracts was highly heritable (Sprooten et al., 2014; Kochunov et al., 2015). Our present results did not show strong correlations of FA among the twin pairs, but this discrepancy may be due to small sample size. Further studies recruiting both monozygotic and dizygotic twins with better tractography analyses would clarify the contribution of genetic and environmental factors to the language-related pathways. Moreover, methodological improvements on thresholding with less-manual adjustments in seed selection are expected to increase the reliability of diffusion MRI analyses. In the present study, we suggest that some task-related skills and word-level knowledge are largely associated with shared factors between twins, whereas individual syntactic abilities in L2 are less prone to shared factors. We also suggest that the thickness of the left Arcuate may be more associated with shared factors than FA was. Our present results further raise an important question regarding how L2 acquisition and general development in juvenile ages are differentially related to the plasticity of the left Arcuate.

## Author Contributions

All authors listed, have made substantial, direct and intellectual contribution to the work, and approved it for publication.

## Conflict of Interest Statement

The authors declare that the research was conducted in the absence of any commercial or financial relationships that could be construed as a potential conflict of interest.

## Footnotes

^1^http://fsl.fmrib.ox.ac.uk/fsl/fslwiki/

^2^https://cran.r-project.org/web/packages/ppcor/

^3^http://www.r-project.org/

^4^https://cran.r-project.org/web/packages/cocor/
